# Supplementary material for: Assessing breast cancer awareness on reproductive age women in West Badewacho Woreda, Hadiyya Zone, South Ethiopia; Community based cross- sectional study
Source: PLoS One. 2022 Jul 27;17(7):e0270248. doi: 10.1371/journal.pone.0270248 (PMC9328547; doi:10.1371/journal.pone.0270248)
Supplement: S1 Data — (DOCX) [file pone.0270248.s001.docx]

# English version questionnaire

**Part I- Socio demographic Characteristics of respondents**

| S. no | Questions | Answer | Remark |
| --- | --- | --- | --- |
| 101 | How old are you? | ….......Years (age in completed years) |  |
| 102 | Residence | 1. Rural 2. Urban |  |
| 103 | Which of the following best describes your main work status? | 1. Farmer  2. Government employee  3. Merchant  4. Daily laborer  5. Housewife  6. Other ; specify______________ |  |
| 104 | What is the highest educational level you attained? | 1. Cannot read and write  2. Read and write(non-formal  3. Primary  4. Secondary  5. Higher or tertiary |  |
| 105 | What is your religion? | 1. Orthodox  2. Catholic  3. Muslim  4. Protestant  5.Others (specify)_____________ |  |
| 106 | To what ethnic group do you belong to? | 1. hadiyya  2. kambata  3. wolayita  4. Gurage  5. Sidama  6. silte  7. Other(specify)_________________ |  |
| 107 | Marital status | 1. Married  2. Single  3. Divorced  4. Widowed  5. Others ; ______________ |  |
| 108 | Monthly Household Income | In ETH Birr___________________ |  |
| 109 | What is the highest educational level your husband attained? | 1. Cannot read and write  2. Read and write(non-formal  3. Primary  4. Secondary  5. Higher or tertiary |  |
| 110 | Do you have family history of breast cancer? | 1. Yes 2. No |  |
| 111 | Do you have TV /radio in your house? | 1. Yes 2. No |  |

**II. General awareness of breast cancer**

| s. no | Questions | Response category | Skip to |
| --- | --- | --- | --- |
| 201 | Have you heard about breast cancer? | 1. Yes 2. No | If Q 201, 0 discontinue interview |
| 202 | If yes Q 201; From where did you hear about breast cancer? | 1. Radio /Television 2. Learned at class 3. Health worker 4. Books /net 5. Friends 6. Neighbors 7. Others (specify) ______ |  |
| 203 | Which sign and symptoms breast cancer can develop? | 1. Breast lump 2. Breast pain 3. Discharge 4. Nipple retraction 5. Redness and engorgement 6. Itching 7. Change in size of the breast |  |
| 204 | What Specific risk factors of breast cancer do you know? | 1. Family history 2. Contraceptive pills 3. Increasing age/aging 4. Being woman 5. Obesity 6. Not breast feeding 7. Early onset of menarche 8. Late menopause 9. Smoking 10. Alcohol |  |
| 205 | Do you think that breast cancer preventable? | 1. Yes 2. No | If Q 205, 0 jump to Q 207 |
| 206 | If yes Q205; By which method can it be prevented? | 1. Initiate Breast feeding 2. No smoking 3. Not drinking alcohol 4. Regular screening 5. Physical Exercise 6. Combat obesity 7. Avoid OCP 8. Wearing bra |  |
| 207 | What common screening methods are of breast cancer do you know? | 1. Breast self examination 2. Clinical breast examination 3. Mammography |  |
| 208 | Is breast cancer is treatable? | 1. Yes 2. No | If Q 208, 0 skip interview |
| 209 | If yes Q 208; What is the treatment of breast cancer? | 1. Chemotherapy and radiotherapy 2. Hormonal therapy 3. Surgery or removal of the whole breast |  |
