# Supplementary material for: Assessing breast cancer awareness on reproductive age women in West Badewacho Woreda, Hadiyya Zone, South Ethiopia; Community based cross- sectional study
Source: PLoS One. 2022 Jul 27;17(7):e0270248. doi: 10.1371/journal.pone.0270248 (PMC9328547; doi:10.1371/journal.pone.0270248)
Supplement: Attachment — Submitted filename: SS Review_ PONE-D-21-11416_reviewer.pdf [file pone.0270248.s002.pdf]

PLOS ONE

ASSESSING BREAST CANCER AWARENESS ON REPRODUCTIVE AGE WOMEN IN WEST BADEWACHO WOREDA, HADIYYA ZONE, SOUTH ETHIOPIA: COMMUNITY BASED CROSS- SECTIONAL STUDY.

--Manuscript Draft--

**Manuscript Number:**	PONE-D-21-11416
**Article Type:**	Research Article
**Full Title:**	ASSESSING BREAST CANCER AWARENESS ON REPRODUCTIVE AGE WOMEN IN WEST BADEWACHO WOREDA, HADIYYA ZONE, SOUTH ETHIOPIA: COMMUNITY BASED CROSS- SECTIONAL STUDY.
**Short Title:**	ASSESSING BREAST CANCER AWARENESS
**Corresponding Author:**	Mengistu Funga Lodebo, MSc<br>Hossana College of Health Sciences<br>hossana, SNNPR ETHIOPIA
**Keywords:**	awareness, cancer, breast, reproductive, Ethiopia.
**Abstract:**	Introduction : Globally, breast cancer is most commonly diagnosed cancer in women, both in the high and low-income countries. Developing individual and community awareness on breast cancer can play a vital role. However, in Ethiopian women the awareness about breast cancer is not well documented. Thus, the aim of this study was to assess the awareness of breast cancer and associated factors among reproductive age women in West Badewacho Woreda, Hadiya Zone, South Ethiopia, 2020.<br><br>Method : A community based-cross sectional study was employed from April 18 to May 16, 2020. Pre-tested structured interviewer administered questionnaire was used to collect data from study participants. Multistage random sampling technique was employed to select reproductive age women from Woreda. Data was entered into a computer using Epi data version 3.1, and edited, cleaned and analyzed using SPSS windows version 20. Binary logistic regression analyses were used to identify determinants related to awareness of breast cancer.<br><br>Result: A total of 578 reproductive-age women were participated in this study. Only 364 reproductive age women (63%) had heard about breast cancer. Out of these, 234 (64.3%) of respondents had aware about breast cancer. After adjusting for potential co-founders, husbands educational status [AOR=0.262; 95% CI (0.076, 0.900)], family history of breast cancer [AOR=0.281; 95% CI (0.132, 0.594)] and having TV/Radio [AOR=0.489; 95% CI (0.246, 0.972)] were significant predictors for awareness of breast cancer.<br><br>Conclusion: This study revealed that it needs to deal with the awareness creation of breast cancer among women in the study area.
**Order of Authors:**	Mengistu Funga Lodebo, MSc
	Zerihun Damissie
	Anebo Getachewu
	Tessema Bereku
**Additional Information:**	
**Question**	**Response**
**Financial Disclosure**<br><br>Enter a financial disclosure statement that describes the sources of funding for the work included in this submission. Review	no

Powered by Editorial Manager® and ProduXion Manager® from Aries Systems Corporation

the submission guidelines for detailed requirements. View published research articles from *PLOS ONE* for specific examples.<br><br>This statement is required for submission and **will appear in the published article** if the submission is accepted. Please make sure it is accurate.<br><br>**Unfunded studies**<br>Enter: *The author(s) received no specific funding for this work.*<br><br>**Funded studies**<br>Enter a statement with the following details:<br>• Initials of the authors who received each award<br>• Grant numbers awarded to each author<br>• The full name of each funder<br>• URL of each funder website<br>• Did the sponsors or funders play any role in the study design, data collection and analysis, decision to publish, or preparation of the manuscript?<br>• **NO** - Include this sentence at the end of your statement: *The funders had no role in study design, data collection and analysis, decision to publish, or preparation of the manuscript.*<br>• **YES** - Specify the role(s) played.<br><br>* typeset	
**Competing Interests**<br><br>Use the instructions below to enter a competing interest statement for this submission. On behalf of all authors, disclose any competing interests that could be perceived to bias this work—acknowledging all financial support and any other relevant financial or non-financial competing interests.<br><br>This statement is required for submission and **will appear in the published article** if the submission is accepted. Please make sure it is accurate and that any funding sources listed in your Funding Information later in the submission form are also	no

declared in your Financial Disclosure statement.<br><br>View published research articles from *PLOS ONE* for specific examples.<br><br>**NO authors have competing interests**<br><br>Enter: *The authors have declared that no competing interests exist.*<br><br>**Authors with competing interests**<br><br>Enter competing interest details beginning with this statement:<br><br>*I have read the journal's policy and the authors of this manuscript have the following competing interests: [insert competing interests here]*<br><br>* typeset	
**Ethics Statement**<br><br>Enter an ethics statement for this submission. This statement is required if the study involved:<br><br>• Human participants<br>• Human specimens or tissue<br>• Vertebrate animals or cephalopods<br>• Vertebrate embryos or tissues<br>• Field research<br><br>Write "N/A" if the submission does not require an ethics statement.<br><br>General guidance is provided below. Consult the submission guidelines for detailed instructions. **Make sure that all information entered here is included in the Methods section of the manuscript.**	human subject research

**Format for specific study types**<br><br>**Human Subject Research (involving human participants and/or tissue)**<br>• Give the name of the institutional review board or ethics committee that approved the study<br>• Include the approval number and/or a statement indicating approval of this research<br>• Indicate the form of consent obtained (written/oral) or the reason that consent was not obtained (e.g. the data were analyzed anonymously)<br><br>**Animal Research (involving vertebrate animals, embryos or tissues)**<br>• Provide the name of the Institutional Animal Care and Use Committee (IACUC) or other relevant ethics board that reviewed the study protocol, and indicate whether they approved this research or granted a formal waiver of ethical approval<br>• Include an approval number if one was obtained<br>• If the study involved *non-human primates*, add *additional details* about animal welfare and steps taken to ameliorate suffering<br>• If anesthesia, euthanasia, or any kind of animal sacrifice is part of the study, include briefly which substances and/or methods were applied<br><br>**Field Research**<br><br>Include the following details if this study involves the collection of plant, animal, or other materials from a natural setting:<br>• Field permit number<br>• Name of the institution or relevant body that granted permission	
**Data Availability**<br><br>Authors are required to make all data underlying the findings described fully available, without restriction, and from the time of publication. PLOS allows rare exceptions to address legal and ethical concerns. See the PLOS Data Policy and FAQ for detailed information.	Yes - all data are fully available without restriction

A Data Availability Statement describing where the data can be found is required at submission. Your answers to this question constitute the Data Availability Statement and **will be published in the article**, if accepted.<br><br>**Important:** Stating 'data available on request from the author' is not sufficient. If your data are only available upon request, select 'No' for the first question and explain your exceptional situation in the text box.<br><br>Do the authors confirm that all data underlying the findings described in their manuscript are fully available without restriction?	
**Describe where the data may be found in full sentences. If you are copying our sample text, replace any instances of XXX with the appropriate details.**<br><br>• If the data are **held or will be held in a public repository**, include URLs, accession numbers or DOIs. If this information will only be available after acceptance, indicate this by ticking the box below. For example: *All XXX files are available from the XXX database (accession number(s) XXX, XXX.).*<br>• If the data are all contained **within the manuscript and/or Supporting Information files**, enter the following: *All relevant data are within the manuscript and its Supporting Information files.*<br>• If neither of these applies but you are able to provide **details of access elsewhere**, with or without limitations, please do so. For example:<br><br>*Data cannot be shared publicly because of [XXX]. Data are available from the XXX Institutional Data Access / Ethics Committee (contact via XXX) for researchers who meet the criteria for access to confidential data.*<br><br>*The data underlying the results presented in the study are available from (include the name of the third party*	xxx

and contact information or URL).<br>• This text is appropriate if the data are owned by a third party and authors do not have permission to share the data.<br><br>* typeset	
Additional data availability information:	Tick here if the URLs/accession numbers/DOIs will be available only after acceptance of the manuscript for publication so that we can ensure their inclusion before publication.

ASSESSING BREAST CANCER AWARENESS ON REPRODUCTIVE AGE WOMEN IN WEST BADEWACHO WOREDA, HADIYYA ZONE, SOUTH ETHIOPIA; COMMUNITY BASED CROSS- SECTIONAL STUDY.

Mengistu Lodebo Funga[1*], Zerihun Damissie[1], Anebo Getachewu[1], Tessema Bereku[2]

[1*]Midwifery Department, Hosanna College of health sciences, Hossana, Ethiopia; [1,2] Midwifery Department, Hosanna College of health sciences, Hossana, Ethiopia;

Correspondence author: Mengistu Lodebo Funga

Department of midwifery, Hosanna College of health science, hosanna, Ethiopia

P.O.Box-159

Tel no: +251920041254/955435735

Fax no: +465552227

Email: mengestulodebo@gmail.com

Abstract

Introduction: Globally, breast cancer is most commonly diagnosed cancer in women, both in the high and low-income countries. Developing individual and community awareness on breast cancer can play a vital role. However, in Ethiopian women the awareness about breast cancer is not well documented. Thus, the aim of this study was to assess the awareness of breast cancer and associated factors among ~~reproductive age~~ women in West Badewacho Woreda, Hadiya Zone, South Ethiopia, 2020.

reproductive-aged women

Method: A community based-cross sectional study was employed from April 18 to May 16, 2020. Pre-tested structured interviewer administered questionnaire was used to collect data from study participants. Multistage random sampling technique was employed to select reproductive age women from Woreda. Data was entered into a computer using Epi data version 3.1, and edited, cleaned and analyzed using SPSS windows version 20. Binary [and multivariable] logistic regression analyses were used to identify determinants related to awareness of breast cancer.

Result: A total of 578 reproductive-age women were participated in this study. Only 364 reproductive age women (63%) had heard about breast cancer. Out of these, 234 (64.3%) of respondents ~~had~~ [were] aware about breast cancer. ~~After adjusting for potential co founders,~~ husbands' educational status [AOR=0.262; 95% CI (0.076, 0.900)], family history of breast cancer [AOR=0.281; 95% CI (0.132, 0.594)] and having TV/Radio [AOR=0.489; 95% CI (0.246, 0.972)] were significant predictors for awareness of breast cancer.

This is not a causal model of analysis. So, it would be better to say "After adjusting for other variables" instead of using the word confounders.

"It" is a vague word here.

Conclusion: This study revealed that it needs to deal with the awareness creation of breast cancer among women in the study area.

Key words: awareness, cancer, breast, reproductive, Ethiopia.

Introduction

Breast cancer is one cancer type, which is a malignant tumor originating from breast tissue, which has glands for milk production, called lobules, and the ducts that connect the lobules to the nipple [1,2].

The specific cause for breast cancer is unknown, but it is considered as a disease mainly associated with some risk factors. Being a female, age, family history of breast cancer especially a first-degree relative, early menarche at the age of 12 and under, late menopause after the age of 55 years, and having the first child after the age of 30 years, prolonged use of oral contraceptive pills, hormonal therapy, and being overweight or obese and null parity are risk factors. But, feeding breast milk appears to be protective against breast cancer [3–5].

Breast cancer is the most frequently diagnosed cancer in women (24.2%, i.e. about one in 4 of all new cancer cases diagnosed in women worldwide are breast cancer), and impacting 2.1 million women each year, and also causes the greatest number of cancer-related deaths among women. In 2019, an estimated 268,600 new cases of invasive breast cancer will be diagnosed among women and 41,760 women are expected to die from breast cancer. While breast cancer rates are higher among women in more developed regions, rates are increasing in nearly every region globally [6,7].

Re-write this sentence

Breast cancer is not only a disease of the developed countries although low and middle-income countries are also affected. In fact, around sixty (58%) of women who die from breast cancer live in developing countries, where the chances of survival can be as low as 20%. Similarly, breast cancer is the first most often occurring among women in Ethiopia. Many cases unreported as women living in rural areas often seek treatment from traditional healers before seeking help

from medical centers[8]. Due to weak health care systems and limited access to early diagnosis and treatment, women in developing countries are likely to be diagnosed only at a late stage of breast cancer and in many settings; even access to supportive and palliative care is lacking [9].

Even though, Ethiopian government investment a lot in the health sector, communicable, non communicable and infectious diseases are still the major public health issues in the country [10].

The poor awareness, wrong beliefs and present for care at a late stage in the disease for breast cancer among women, where made them treatment is most in effective, negative perception of the curability of a cancer detected early [3,11].

Promotion of women's awareness about breast cancer and its symptoms can encourage them to perform better and timelier breast self-exams but lower awareness about breast cancer in rural women reduces their chance of detecting breast cancer symptoms, causing the breast cancer be

Please re-write the sentence to give a clear sense.

detected at an advanced stage [12]. Similarly, in Ethiopian community where delayed presentation is predominant and majorly patients are diagnosed at advanced stages there is improving the levels of awareness about breast cancer needs urgently. However, breast cancer awareness is not well documented in Ethiopian women. Therefore, the aim of this study was to assess awareness of breast cancer and associated factors among reproductive age women in Hadiya Zone, West Badewacho Woreda, 2020.

Materials and methods

Study design, setting and sampling

A community based cross sectional study design was employed in West Badewacho woreda, which is found in Hadiyya Zone, SNNPR. The sample size of this study was calculated using single population proportion formula taking proportion of awareness/ knowledge of breast cancer in Bale Zone [11], margin of error, confidence level, design effect and non response rate ~~were~~ [Please justify why the author used the 1.5 design effect.] assumed to be 56.2%, 5%, 95% ,1.5 and 5 %, respectively.

$$n = \frac{(Z\alpha/2)^2 \, p\,(1-p)}{d^2}$$

Where: ni= Sample size; Z $(\alpha/2)^2$ = confidence level; p = proportion of awareness (0.562); d = marginal of error

$$n = \frac{(1.96)^2 \,(0.562)\,(1-0.562)}{(0.05)^2}$$

n = 378 Individuals Using design effect 1.5 i.e. 378*1.5=567 Thus, finally taking 5 % non-response rate the final sample size was **595**.

A multistage random sampling technique was employed. Initially, stratification of woreda [into] ~~in to~~ urban and rural, [was done] then all the rural and urban Kebeles in the woreda was listed separately in the frame. Then, seven rural and one urban Kebeles [were] ~~was~~ selected randomly using lottery method. Proportionally allocation was done based on the size of households consisting eligible population to each selected kebeles. [Please check and re-write to make it more clear.] Again list of gotts in each randomly selected kebeles. Then using

lottery methods, selection of the gotts in each randomly selected kebeles were done. Again list of households containing eligible reproductive age women in selected kebele at each gott level was extracted from the health extension works as a sampling frame. Again proportionally allocation was done based on the number of households consisting eligible population to each selected gott from each selected kebele. Then, using simple random method was applied to select households containing eligible reproductive age women from sampling frame. Then after data collectors went to the household by using head of house hold name with guidance of health extension workers. Finally, Lottery method was used to select one woman interviewee whenever two or more women of child bearing age (18–49 years) exist in the selected household**.**

Please check and re-write to make it more clear.

Data collection method

Pretested structured questionnaire was used to collect data from each study participant~~s.~~ The questionnaire was adapted from related literatures [9,11,13] with minor modification in line with the objectives of this particular study and to fit to the local context.

Seven diploma nurses were recruited as data collector while three BSc nurses were recruited as supervisors. The principal investigator would follow overall data collection procedure plan.

Face to face interviewer administered method of data collection was employed using Ahmaric version structured questionnaire.

Operational definition and terms

- **Awareness of breast cancer**- was assessed by asking questions related to awareness about breast cancer (risk factor, sign &symptoms, preventive measures, screening methods and treatment). Each correct answer was scored 'Yes' and each incorrect answer were scored 'No'. Then, the total scores were ranked. Re-write to make it more clear. Those reproductive age group women score equal to mean and above defined high score and hence it was labeled as **knowledgeable/aware** and those reproductive age group women score less than mean defined as a low score it was labeled as **not knowledgeable/ un aware** for the awareness of breast cancer [13,14].

Data Quality Assurance

Two-day ~~Two days~~ training was provided to the data collectors and supervisors. The questionnaire was first prepared in English, translated into Ahmaric and then it was re-translated back to English to

check for its consistency. Before a week of actual data collection investigator, supervisors and data collectors ~~were~~ took a 5% (30 questionnaire) to pretest ~~of~~ the Ahmaric version of questionnaire in East Badewacho Woreda Ajeba and Wera Lalo kebeles similar characteristics to the study population to ensure clarity of the questionnaire and then the necessary modifications and correction was made to standardize and ensure its validity. Rewrite this sentence to make it clear Daily supervision was done. In addition, the data was thoroughly cleaned and carefully entered into ~~in to~~ computer for beginning of analysis.

Data Processing and Analysis

The collected data was coded and entered into Epi-data version 3.1 and finally exported to SPSS version 20 statistical software for analysis. Recoding and computing of variables were done as needed. To summarize the data, descriptive statistic such as frequency, percentage, mean, standard deviation and range was used. Use “Bivariable analysis” or “Binary logistic regression” Bivariate analysis was done. All variables in bivariate analysis having P-value < 0.25 Please clarify why the author used the p-value of 0.25 instead of 0.05? was candidate to multivariable logistic regression. Back ward likelihood ratio of logistic regression Please elaborate on why the author used this method “Back ward likelihood ratio of logistic regression”. were performed to identify the factors associated with breast cancer awareness. The model goodness fit was checked by Hosmer-Lemeshow test and the p- value was found to be 0.723 (>0.05), which revealed ~~as~~ the model was good. Adjusted Odds ratios (AOR) at 95% CI were computed to measure the strength of the association between the outcome and the explanatory variables. P-value < 0.05 was considered as statistically significant in the study. in the multivariable model.

Ethical Consideration

Ethical clearance and permission was obtained from the Institutional Review Board (IRB) *(code 502/2012 E.C)* of Hosanna College of Health sciences with protocol reference number by *HCHS 09/4452* and offered to Woreda Health Office, and then permission was obtained from study Kebeles. Informed oral consent was obtained after thorough explaining about the study, including purpose, expectations regarding participation, foreseeable risks and benefits from each participant. Voluntary basis participation and responses were was kept confidential and anonymous. Women were interviewed at their home in private area and no family members were ~~are~~ allowed to accompany the participants to permit freedom of expression, privacy, and confidentiality.

RESULTS

From a total of five hundred ninety-five reproductive-age women, 578 reproductive-age women ~~have~~ completed the interview which gave a response rate of 97%. Seventeen incomplete questionnaires were excluded from the analysis. The results are presented under subheadings as follows:

Socio-demographic characteristics of respondents

In this study, the age of participants ranged from 18 to 48 years. The mean age of respondents was 27.59 ± 7.18 SD years. One hundred eighty-two (31.5%) of study participants belonged ~~belong~~ to the age below 23 years. Four hundred sixty-six (80.6%) of participants were rural. Regarding work status 169 (29.2%) were farmers. One hundred seventy-nine (31%) of the women Could not ~~cannot~~ read and write. Regarding religion, three fourth 434 (75.4%) of respondents were protestant religious followers. Five hundred forty-two (93.8%) of the respondents were Hadiya by ethnicity.

Regarding marital status, ~~near to~~ Nearly two-third 368 (63.7%) of women were married. One hundred fifteen (31.3%) of respondents' husbands' educational status were cannot read and write. Three to fourth 434(75.4%) respondents were monthly income below 1311 ETB that was $ 1.90. [Please re-write these sentence to make them clear.] Only 103 (17.8%) of respondents reported that they had a family history of breast cancer. Around thirty-seven percent of study participants had TV/Radio **[table 1]**.

General awareness of breast cancer

From the total of respondents, 364 reproductive age women (63%) had heard about breast cancer. According to the operational definition, 130 (35.7%) of the respondents had were unaware/not knowledgeable i.e. scored less than the mean (4.39 ±SD 4.27). Whereas, 234(64.3%) of respondents had aware/knowledgeable i.e. scored greater than or equal to the mean [Re-write to make it clear.] [**Figure 1**].

Breast lump was mentioned by the majority (60.4%) of the women as being ~~of~~ signs and symptoms of breast cancer. Nearly half (51.1%) of respondents have had reported family history as a common risk factor for breast cancer. The majority (64.6%) of participants mentioned breast cancer was preventable and they ~~were~~ reported regular screening (53.6%) as a common preventive method of breast cancer. Among those who heard about breast cancer more than three fourth (77.5%) of the ~~study,~~ participants mentioned clinical breast examination as a common screening method for breast cancer and 59.1% of respondents believe believed that breast cancer was treatable. More than half (68.4%) participants mentioned hormonal therapy as a major treatment method [**Table 2**].

Sources of information

From those totally heard about breast cancer, one hundred eighty-seven (51.4%) heard from health workers, neighbors 138(37.9%), and friends 133 (36.5%). Also, 124(34.1%) from radio/TV, 75(20.6%) learned at class, 55(15.1%) from books/nets, and 16(4.4%) from others like internet and magazine [**Figure 2**]. Please re-write this sentence to make it clear.

Factors associated with awareness of breast cancer

Please re-write this sentence to make it clear. bivariable

Also , be consistent with the use of the p-value of 0.25 or 0.05, as line 366 of Table 3. these

Seven independent variables were identified during the bivariate logistic regression analysis, which has a p-value of less than 0.25. Among this variables, residence, the work status of women, educational level of the women, educational level of the husband, monthly income, family history of breast cancer, and having TV/Radio has a crude association with an awareness of breast cancer [**Table 3**]. had

Multivariate analysis Multivariable Analysis

Check consistency with use of 0.25/0.05 bivariable

All variables with a p-value of less than 0.25 during bivariate analysis were entered into multivariate logistic regression analysis. The variables with a p-value less than 0.05 in multivariate analysis were considered as statistically significant in the study. The variables, which significantly predict awareness of breast cancer, include the educational status of the husband, family history of breast cancer, and having TV/Radio.

Line 201-203: It would be better to include these sentences in the Data Analysis Section. predicted

In multivariate logistic regression analysis, those whose husbands attended primary school were 74% less likely to be aware of breast cancer compared to those whose husbands attended tertiary school [AOR=0.262; 95% CI (0.076, 0.900)]. Similarly, those who had no family history of breast cancer were 72% less likely to be aware of breast cancer compared to those who had a family history of breast cancer [AOR=0.281; 95% CI (0.132, 0.594)]. In the same way, those

In this section, as well as in the Tables, two significant digits after the decimal point are enough.

who had no TV/Radio were 51% less likely to be aware of breast cancer compared to their counterparts [AOR=0.489; 95% CI (0.246, 0.972)] [**table 4**].

Discussion

Improved

Improve awareness of the society about non-communicable diseases especially breast cancer and its predictors is important for the success of prevention intervention. So, this study ~~was~~ assessed awareness of breast cancer and associated factors among ~~r~~reproductive-age women.

The finding of this study showed that 64.3% of the study participants were aware of breast cancer from the composite score regarding signs and symptoms, risk factors, method of prevention, common diagnosis methods, and treatment of breast cancer. This study was consistent with the study conducted by Iran [12], central India [14], Nigeria [3] and Ethiopia[15]. This finding is higher than the cross-sectional study conducted in Eastern China ~~eastern china~~ (18.6%) [16] and Saudi Arabia (39.7%) [17]. This difference might be due to sample size, respondent's inclusion criteria, and data collection methods, total number of items they were used to ask the respondents, construction and compute of the awareness item.

Please re-write this sentence to make it clearer.

The author could specify one or two instances of how the different methodological aspects like sample size or total number of items used to ask respondents could have led to lower prevalence of breast cancer awareness in Eastern China (18.6%) and Saudi Arabia (39.7%) , as compared to this study (64.3%).

Also finding of this study is lower than the study conducted in Benin (92.6%) [18]. This could most probably be due to sample size, socio-cultural characteristics of respondents and cut point score of the item for awareness. Also the participants were health education during repeated visit of the health facility due immunize the child. This sentence does not give a clear meaning. Please re-write.

Those whose husbands attended primary school were less likely to be aware of breast cancer compared to those whose husbands attended tertiary school. This finding was supported by a study finding on Turkey ~~Turkish~~ [19] and China china [20]. This could most probably be naturally expected

that people having higher levels of education have more capability for obtaining more and effective information from various sources and they have the opportunity to communicate with their families on these specific health problems. Also, they have more capability to equip their home with different information sources and better income to avail it.

This study finding showed that a family history of breast cancer is associated with an awareness of breast cancer. Those who had no family history of breast cancer were less likely to be aware than their counterparts. This finding was similar to a study conducted in Iran [21], China [16], and Ethiopia [15]. This might be ~~due to~~ because a family history of breast cancer gave them a chance to have information and ~~increases~~ increased health-seeking behavior.

The present study found a significant association between having TV/Radio and awareness of breast cancer. This finding was supported by findings in Nigeria [22,23] and Uganda [24]. This might be the mass media play a key role in the development process of a country. [Please re-write to make the sentence clear.] The media are generally regarded as channels of communication that are capable of reaching diverse audiences concurrently with the same messages. Also, they persuade and catalyze social mobilization. In other words, the mass media can be regarded as powerful sources of information because they have the capability to penetrate every segment of the community. They have capable [capacity] to disseminate messages about issues, ideals, and products. Additionally, the mass media have the capacity to create awareness and knowledge about issues of wanted interest. Therefore, different scholar's widely agreed that awareness leads to knowledge, and knowledge leads to behavior modification .

Conclusion

Rewrite this sentence to make it clear.

This study focused that needs to deal with the awareness creation of breast cancer among women in the study area. Husband's educational status, family history of breast cancer, and having TV/Radio were significantly ~~statistically~~ associated with an awareness of breast cancer. Better to improve health service programs that be capable of touch all women according to their favorable area and opportunities to increase awareness of breast cancer through health programs in form of the media coverage through newspapers, local written and oral, radio and television regarding its sign and symptoms, risk factors, early detection, prevention and management systems to decrease the weight of breast cancer.

This sentence does not give a clear sense. Please re-write.

Also better to use cancer literacy programs at nation and state wide, as well as engagements with community- level organizations and all level health delivery systems.

Acknowledgements

Our deepest gratitude to Hosanna college of Health Sciences, research and publication directorate director particularly for research review board for facilitation of this research work.

Also, we would like to express our thanks to Woreda Health Office, study participants, supervisor and data collectors for their co-operation during data collection.

Lastly, we would like to express our thanks to our advisor for his continuous comments, advice and suggestion.

Conflict of interests

The authors declare that they have no conflict of interests.

Funding statement

Hosanna College of health science funded this research but the funding organization has no effect on the scientific procedure of this paper.

Data availability

The datasets used during the current study are available from the corresponding author on reasonable request.

References

1. Wurjine TH, Menji ZA, Bogale N. Assessment of knowledge, attitude and practice towards breast cancer early detection methods among female health professionals at public health centers of Addis Ababa, Ethiopia, 2017. Women's Heal. 2019;8(3):201–2019.

2. Amosu AM, Degun AM, Thomas AM, Babalola AO. Assessment of awareness, perception, specific knowledge, and screening behaviour regarding breast cancer among rural women in Ipokia Local Government Area, Ogun State, Nigeria. Arch Appl Sci Res [Internet]. 2011;3(2):253–65. Available from: http://scholarsresearchlibrary.com/aasr-vol3-iss2/AASR-2011-3-2-253-265.pdf

3. Omotara B, Yahya S. Awareness, Attitude and Practice of Rural Women regarding Breast Cancer in Northeast Nigeria. J Community Med Health Educ. 2012;2(5):2–5.

4. Hashim I. Principles and Practice of Cancer Prevention and Control Redhwan Ahmed Al-Naggar Cervical Cancer : Prevention and Control. 2015;(December):1–12.

5. B A, W Y, W J, Y B, H A, W B, et al. Breast cancer awareness among female school

teachers in Makkah region, Saudi Arabia. A cross-sectional study. Glob Surg. 2019;5(2):1–5.

6. WHO. Latest global cancer data : Cancer burden rises to 18 . 1 million new cases and 9 . 6 million cancer deaths in 2018 Latest global cancer data : Cancer burden rises to 18 . 1 million new cases and 9 . 6 million cancer deaths in 2018. Press release. 2018;(September):13–5.

7. Society AC. Breast Cancer. Breast Cancer Facts Fig 2019-2020. 2020;1–32.

8. Gemta EA, Bekele A, Mekonen W, Seifu D, Bekurtsion Y, Kantelhardt EJ. Patterns of Breast Cancer Among Ethiopian Patients: Presentations and Histopathological Features. J Cancer Sci Ther. 2019;11(2):38–42.

9. Kumar M, Kashyap V. Awareness about breast cancer among women attending obstetrics and gynaecology department in a tertiary care hospital of Jharkhand, India. Int J Community Med Public Heal. 2016;3(4):938–43.

10. Abay M, Tuke G, Zewdie E, Abraha TH, Grum T, Brhane E. Breast self-examination practice and associated factors among women aged 20-70 years attending public health institutions of Adwa town, North Ethiopia. BMC Res Notes [Internet]. 2018;11(1):1–7. Available from: https://doi.org/10.1186/s13104-018-3731-9

11. Hussen A, Kumbi M, Abate L, Nuriye S. Knowledge of Breast Cancer and Associated Factors among Women Reproductive Age in Bale Zone, Southeast Ethiopia: A Community based Cross Sectional Study. 2019;9(2).

12. Naqvi AA, Zehra F, Ahmad R, Ahmad N, Yazdani N, Usmani S, et al. Awareness,

knowledge and attitude towards breast cancer, breast screening and early detection techniques among women in Pakistan. J Pak Med Assoc. 2018;68(4):576–86.

13. Abeje S, Seme A, Tibelt A. Factors associated with breast cancer screening awareness and practices of women in Addis Ababa, Ethiopia. BMC Womens Health. 2019;19(1):1–8.

14. Gangane N, Ng N, Sebastián MS. Women's knowledge, attitudes, and practices about breast cancer in a Rural District of Central India. Asian Pacific J Cancer Prev. 2015;16(16):6863–70.

15. Lemlem SB, Sinishaw W, Hailu M, Abebe M, Aregay A. Assessment of Knowledge of Breast Cancer and Screening Methods among Nurses in University Hospitals in Addis Ababa, Ethiopia, 2011. ISRN Oncol. 2013;2013:1–8.

16. Liu LY, Wang F, Yu LX, Ma ZB, Zhang Q, Gao DZ, et al. Breast cancer awareness among women in Eastern China: A cross-sectional study. BMC Public Health. 2014;14(1):1–8.

17. Al-Suroj HR, Al Ali FB, Al-Saleh KH. Awareness and Attitude among Saudi Females toward Breast Cancer Screening in Al-Ahsa, KSA. Egypt J Hosp Med. 2018;71(2):2516–22.

18. Ifeanyichukwu O. Assessment of Breast Cancer Screening Practices among Women of Reproductive Age in Benin City, Edo State. Int J Trop Dis Heal. 2015;6(4):141–9.

19. El Mhamdi S, Bouanene I, Mhirsi A, Sriha A, Ben Salem K, Soltani MS. Women's knowledge, attitudes and practice about breast cancer screening in the region of Monastir (Tunisia). Aust J Prim Health. 2013;19(1):68–73.

20. Liu N, Li P, Wang J, Guo P, Zhang X, Yang S, et al. Factors influencing breast cancer awareness : a cross-sectional study in China. 2020;

21. Tazhibi M, Feizi A. Awareness Levels about Breast Cancer Risk Factors , Early Warning Signs , and Screening and Therapeutic Approaches among Iranian Adult Women : A large. 2014;2014.

22. Nelson O. Creating Awareness of Breast Cancer. 2014;(September 2011).

23. Nelson O. Effective Use of Media Awareness Campaigns for Breast Cancer Care among Women : A Comparative Study. 2016;17(4):160–73.

24. Atuhairwe C, Amongin D, Agaba E, Mugarura S, Taremwa IM. The effect of knowledge on uptake of breast cancer prevention modalities among women in Kyadondo County , Uganda. 2018;1–8.

Tables

Table 1-Socio-demographic characteristic of respondents in West Badewacho Woreda, Hadiya Zone, South Ethiopia, 2020 (n=578)

Variables	Categories	Frequency	Percentage
**Age**	=<23	182	31.5
	24-29	178	30.8
	30-34	101	17.5
	=>35	117	20.2
	Total	578	100.0
**Residence**	Rural	466	80.6
	Urban	112	19.4
	Total	578	100.0
**Work status**	Farmer	169	29.2
	Housewife	95	16.4
	Student	122	21.1
	Merchant	85	14.7
	Daily laborer	33	5.7
	Gov't employee	74	12.8
	Total	578	100.0
**Educational level of women**	Check the case of the words cannot read and write	179	31.0
	read and write	131	22.7

	Primary	109	18.9
	Secondary	84	14.5
	higher or tertiary	75	13.0
	Total	578	100.0
**Religion**	Orthodox	60	10.4
	Catholic	67	11.6
	Muslim	3	.5
	Protestant	434	75.1
	Others*	14	2.4
	Total	578	100.0
**Ethnicity**	Hadiya	542	93.8
	Kambata	18	3.1
	Wolayita	11	1.9
	Others**	7	1.2
	Total	578	100.0
**Marital status**	Married	368	63.7
	Single	188	32.5
	Divorced	12	2.1
	Widowed	10	1.7
	Total	578	100.0
**Educational level of husband**	Check the case of the words cannot read and write	115	31.3
	read and write(non-formal)	98	26.6

	Primary	57	15.5
	Secondary	36	9.8
	higher or tertiary	62	16.8
	Total	368	100.0
**Income**	1311	434	75.1
	>1311	144	24.9
	Total	578	100.0
**Family history of BC**	No	474	82
	Yes	104	18
	Total	578	100.0
**Having TV/Radio**	No	362	62.6
	Yes	216	37.4
	Total	578	100.0

Others: only Jesus*, Adventist*, Ahmara**, Silte**, Gurage**

TABLE-2.GENERAL AWARENESS ABOUT BREAST CANCER IN WEST BADEWACHO WOREDA, HADIYA ZONE, SOUTH ETHIOPIA, 2020

Variables	Categories	Frequency	Percentage
**Sign and symptoms**	Breast lump	220	60.4
	Breast pain	202	55.5
	Discharge	139	38.2
	Nipple retraction	68	18.7
	Redness and	72	19.8

	engorgement		
	Itching	110	30.1
	Change in size of the breast	37	10.2
**Risk factors**	Family history	186	51.1
	Contraceptive pills	93	25.5
	Increasing age/aging	38	10.4
	Being a woman	162	44.5
	Obesity	23	6.3
	Not breast feeding	71	19.5
	Early-onset of menarche	48	13.2
	Late menopause	43	11.8
	Smoking	44	12.1
	Alcohol	49	13.5
**Breast cancer preventable (n=364)**	No	129	35.4
	Yes	235	64.6
**By which method can it be prevented?**	Initiate breast feeding	118	50.2
	No smoking	61	26
	Not drinking alcohol	58	24.7
	Regular screening	126	53.6
	Physical Exercise	13	5.5

	Combat obesity	9	3.8
	Avoid OCP	34	14.5
	Wearing bra	34	14.5
**What common screening methods are of breast cancer do you know? (n=364)**	Breast self-examination	168	45.3
	Clinical breast examination	283	77.5
	Mammography	40	11
**Is breast cancer ~~is~~ treatable? (n=364)**	No	149	40.9
	Yes	215	59.1
**What is the treatment of breast cancer?**	Chemotherapy and radiotherapy	57	26.5
	Hormonal therapy	147	68.4
	Surgery or removal of the whole breast	135	62.8
***more than one option is reported by a participant.**			

We have asterisk sign in the footnote, but not anywhere in the table.

Bivariable or Binary Logistic Regression

TABLE 3 BIVARIATE ANALYSIS OF BREAST CANCER AWARENESS AND ASSOCIATED FACTORS AMONG REPRODUCTIVE-AGE WOMEN IN WEST BADEWACHO WOREDA, HADIYA ZONE, SOUTH ETHIOPIA, 2020 (N=364)

The author can show the information in Table 3 and Table 4 (Crude Odds Ratio and Adjusted Odds Ratio) in a single table for better presentation and clarity.

Variables	Categories	Aware n (%)	Unaware n (%)	COR(95%CI)	P-value

**Residence**	Rural	169(67.1)	83(32.9)	1.472(0.931,2.328)	0.098
	Urban	65(58)	47(42)	1	
**Work status**	Farmer	37(50.7)	36(49.3)	0.131(0.055, 0.311)	<0.001*
	Housewife	31(56.4)	24(43.6)	0.164(0.066, 0.407)	<0.001*
	Student	47(52.2)	43(47.8)	0.139(0.060, 0.323)	<0.001*
	Merchant	39(70.9)	16(29.1)	0.310(0.121, 0.791)	0.014*
	Daily laborer	17(85.0)	3(15.0)	0.720(0.172, 3.010)	0.652
	Gov't employee	63(88.7)	8(11.3)	1	
**The educational level of women**	cannot read and write	50(60.2)	33(39.8)	0.366(.176, .759)	0.007*
	read and write	56(65.1)	30(34.9)	0.451(.216, .938)	0.033*
	Primary	37(59.7)	25(40.3)	0.357(.165, .774)	0.009*
	Secondary	33(54.1)	28(45.9)	0.284(.132, .615)	0.001*
	higher or tertiary	58(80.6)	14(19.4)	**1**	
**The educational level of**	cannot read and write	29(64.4)	16(35.6)	0.333(0.130,0.850)	0.021*
	read and	35(53)	31(47)	0.207(0.088,0.490)	<0.001*

**husband**	write(non-formal)				
	Primary	13(41.9)	18(58.1)	0.133(0.048,0.363)	<0.001*
	Secondary	15(53.6)	13(46.4)	0.212(0.076,0.592)	0.003*
	higher or tertiary	49(84.5)	9(15.5)	**1**	
**Income**	1311	140(57.9)	102(42.1)	0.409(0.250,0.669)	<0.001*
	>1311	94(77)	28(23)	**1**	
**Family history of BC**	No	151(58.1)	109(41.9)	0.351(0.205,0.601)	<0.001*
	Yes	83(79.8)	21(20.2)	**1**	
**Having TV/Radio**	No	91(52.9)	81(47.1)	0.385(0.248,0.599)	<0.001*
	Yes	143(74.5)	49(25.5)	**1**	

Note: * p value of less than 0.05 in bivariate analysis, '1' reference group

Multivariable

TABLE 4-MULTIVARIATE ANALYSIS OF BREAST CANCER AWARENESS AND ASSOCIATED FACTORS AMONG REPRODUCTIVE-AGE WOMEN IN WEST BADEWACHO WOREDA, HADIYA ZONE, SOUTH ETHIOPIA, 2020 (N=364)

Variables	Categories	COR	AOR	P-value
**The educational level of husband**	cannot read and write	0.3330(0.130,0.850)	0.944(0.279,3.196)	0.927
	read and write(non-	0.207(0.088,0.490)	0.351(0.122,1.008)	0.052

Line 366- The author had described in the Method Section (Line 142) that in the bivariable analysis, they used the p-value of 0.25. In the Line 366, they indicate p value of 0.05. Please apply consistent methods. Also check the appropriate use and label of "*", since this sign has also been used in "<0.001*".

	formal)			
	Primary	0.133(0.048,0.363)	0.262(0.076,0.900)*	0.033
	Secondary	0.212(0.076,0.592)	0.356(0.106,1.167)	0.088
	higher or tertiary	**1**	1	
**Family history of BC**	No	0.351(0.205,0.601)	0.281(0.132,0.594)*	0.001
	Yes	**1**	1	
**Having TV/Radio**	No	0.385(0.248,0.599)	0.489(0.246,0.972)*	0.041
	Yes	**1**		

Note: * statistically significant association in multivariate analysis, “1” reference group

multivariable

Abbreviations

CI: Confidence interval **SPSS**: Statistical package for social sciences **BSE**: Breast Self Examination **CBE**: Clinical Breast Examination

Figures

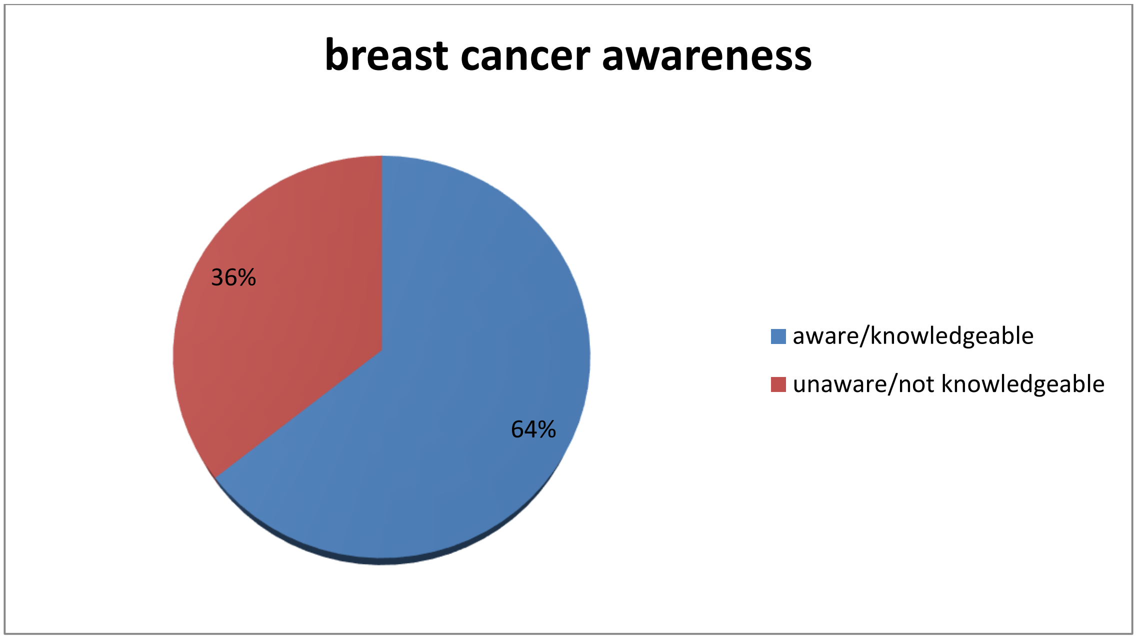

FIGURE 2 AWARENESS OF BREAST CANCER AMONG REPRODUCTIVE-AGE WOMEN IN WEST BADEWACHO WOREDA, HADIYA ZONE, SOUTH ETHIOPIA,2020 (N=364).

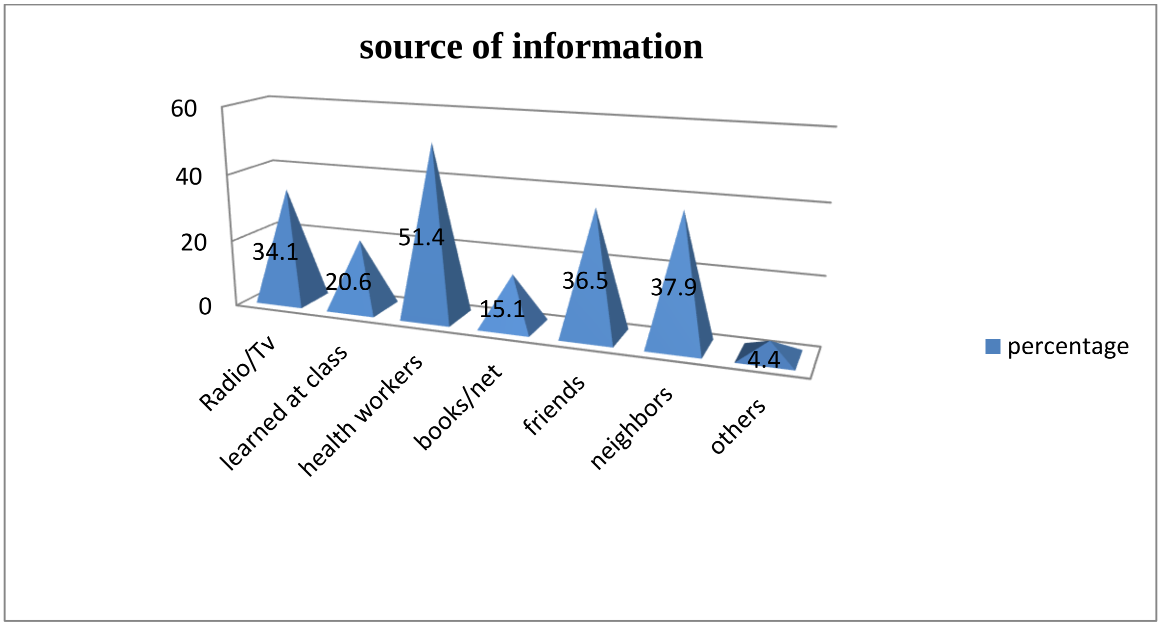

FIGURE 3 SOURCE OF INFORMATION FOR THE AWARENESS OF BREAST CANCER AMONG REPRODUCTIVE-AGE WOMEN IN WEST BADEWACHO WOREDA, HADIYA ZONE, SOUTH ETHIOPIA, 2020.

 Click here to access/download;Figure;figures of breast cancer.pptx

General awareness of breast cancer

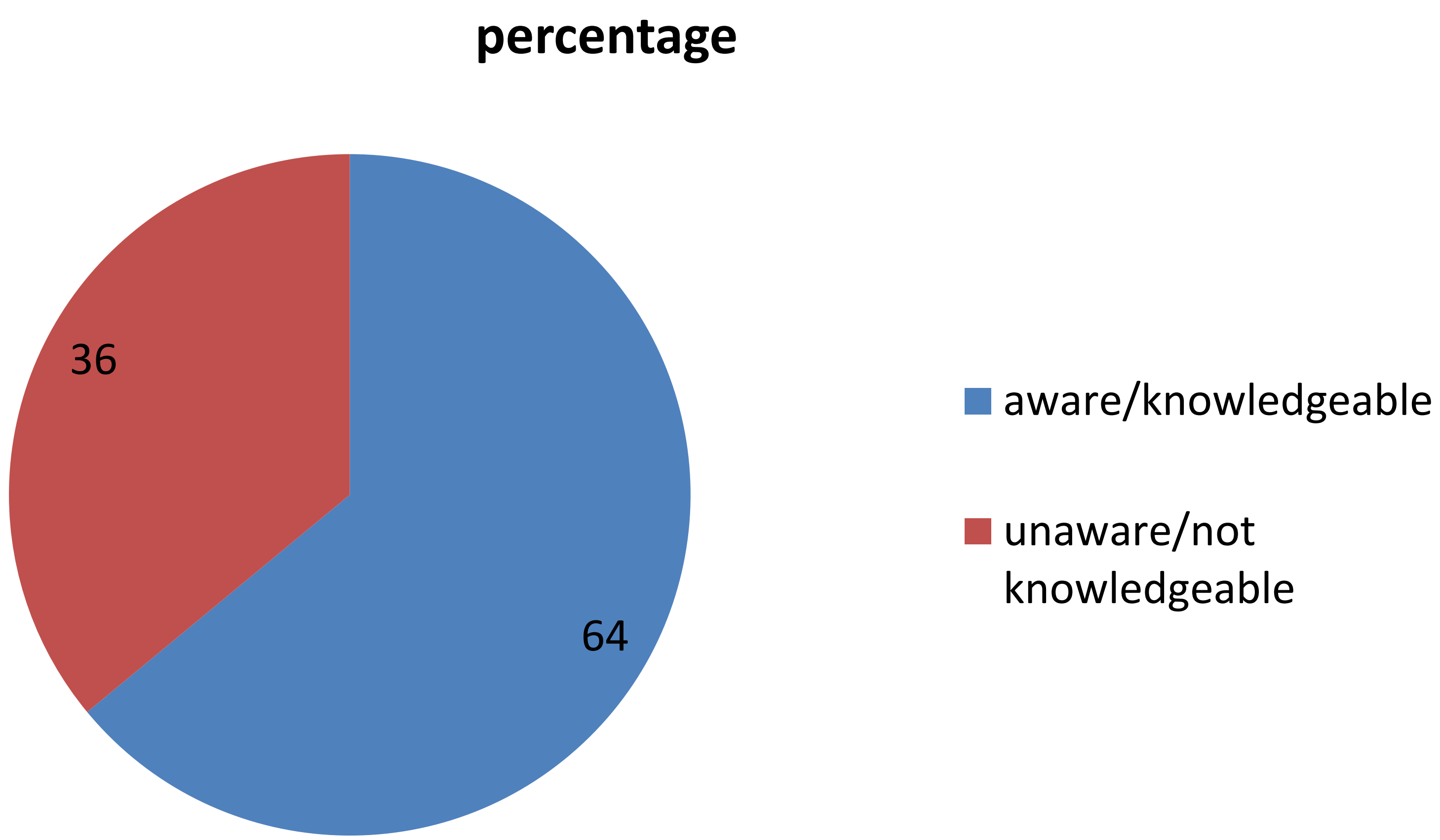

Sources of information

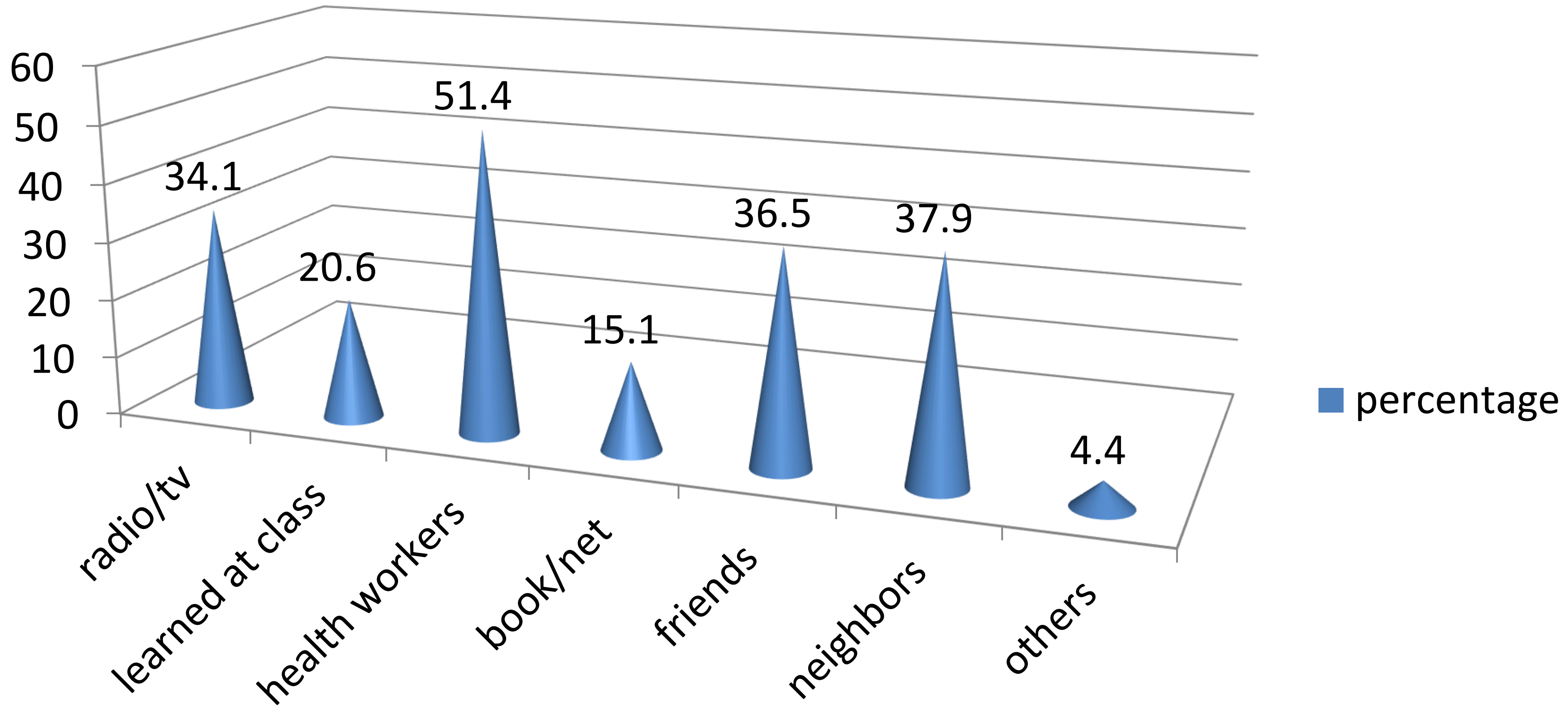

Click here to access/download
Supporting Information
English version questionnaire.docx
