# Supplementary material for: Assessing breast cancer awareness on reproductive age women in West Badewacho Woreda, Hadiyya Zone, South Ethiopia; Community based cross- sectional study
Source: PLoS One. 2022 Jul 27;17(7):e0270248. doi: 10.1371/journal.pone.0270248 (PMC9328547; doi:10.1371/journal.pone.0270248)
Supplement: Attachment — Submitted filename: SS Recommendations_ PONE-D-21-11416_reviewer.docx [file pone.0270248.s003.docx]

**ASSESSING BREAST CANCER AWARENESS ON REPRODUCTIVE AGE WOMEN IN WEST BADEWACHO WOREDA, HADIYYA ZONE, SOUTH ETHIOPIA: COMMUNITY BASED CROSS- SECTIONAL STUDY**

Thank You for the opportunity to review your paper.

# GENERAL COMMENTS

- The author states that since breast cancer awareness is not well-documented among Ethiopian women, this study assesses awareness of the breast cancer and associated factors among reproductive-aged women in Hadiya Zone, West Badewacho Woreda of Ethiopia.
- The study shows that factors like husband’s education status, family history of breast cancer and having TV/Radio were significantly associated with awareness of breast cancer among reproductive-aged women.
- There is not a strong case made for the need for the study. Also, the authors are making a broad assumption of the Ethiopian situation without any references.
- Some of the methodological parts have not been clearly described, and there are some inconsistencies, as suggested in the “Materials and Methods” section below.
- Some elaborations and clarifications can be made in the Discussion section , as suggested in the “Discussion” section below.
- Some of the Recommended Items from the Strobe Checklist have not been illustrated.
  - Variables in the Methods Section
  - Limitations and Interpretations in the Discussion Section
- There are multiple problems with language, grammar and sentence structure, sometimes making the sentences difficult to comprehend. I have indicated many of them in the PDF as well, but there might be additional issues. It is suggested to re-write the manuscript considering language and sentence structure issues.
- Some of the issues are:-
  i. Missing the use of apostrophe mark.
  ii. Missing the use of comma.
  iii. Using present tense instead of past tense in different sections.
  iv. Please use “reproductive-aged women” , instead of reproductive age women in all the sections.
  v. Line 33- Use “were aware” instead of “had aware” .
  vi. Line 57- Please re-write the sentence “Breast cancer is not only a disease of the developed countries although low and middle-income countries are also affected.”
  vii. Line 73-75- Please re-write the sentence to give a clear sense.
  viii. Line 94- use “into” instead of “ in to” . Also sentence not sensible, add “was done”.
  ix. Line 96- Use “were” instead of “was”.
  x. Line 98- Please check and re-write to make it more clear. .
  xi. Line 104- 105- Please check and re-write to make it more clear.
  xii. Line 111- Use “participant” instead of “participants”.
  xiii. Line 122-126- Rewrite this sentence to make it more clear.
  xiv. Line 128- Use “two-day” instead of two days.
  xv. Line 131- Please delete “were”.
  xvi. Line 131- Use “to pretest” instead of “pretest of ” .
  xvii. Line 132-134- Rewrite this sentence to make it clear .
  xviii. Line 135- Use “into” instead of “in to” .
  xix. Line 155- Use “were” instead of “was” .
  xx. Line 156- Use “were” instead of “are” .
  xxi. Line 160 - Delete “have”. Write in past tense.
  xxii. Line 165- Use “belonged” . Write in past tense.
  xxiii. Line 167- Use “ could not” instead of cannot. Write in past tense.
  xxiv. Line 170- Use “ nearly” instead of “near to” .
  xxv. Line 171-172- Please re-write the sentence to make it clear.
  xxvi. Line 177- Use “were” instead of “had” .
  xxvii. Line 178-179- Rewrite to make it clear.
  xxviii. Line 180- Delete “of” .
  xxix. Line 181- Use “had” instead of “have” .
  xxx. Line 183- Delete “were” .
  xxxi. Line 185- Delete “study,” .
  xxxii. Line 186- Use “believed” instead of “believe” .
  xxxiii. Line 187 – Use “more than half” instead of “more half” .
  xxxiv. Line 190 – Use “ From those who totally heard about breast cancer” instead of “From those totally heard about breast cancer” .
  xxxv. Line 191-193- Please re-write this sentence to make it clear.
  xxxvi. Line 195-196- Please re-write this sentence to make it clear.
  xxxvii. Line 196- Use “these” instead of “this”. Also comma is missing
  xxxviii. Line 198- Use “had” instead of “has” .
  xxxix. Line 204- Use “predicted” instead of “predict” .
  xl. Line 215- Delete “was” .
  xli. Line 221- Use “Eastern China” instead of “eastern china”.
  xlii. Line 222- 224- Please re-write this sentence to make it clearer.
  xliii. Line 227-228- This sentence does not give a clear meaning. Please re-write.
  xliv. Line 231- Use “Turkey” instead of “Turkish”. Capitalize “China”.
  xlv. Line 238- Use “counterparts” instead of “counterpart”.
  xlvi. Line 239- 240- Use “because” instead of “due to”, and “increased” instead of “increases”.
  xlvii. Line 243- Please re-write this sentence to make it clearer.
  xlviii. Line 247- Use “capacity” instead of “capable” .
  xlix. Line 251- Use full stop at the end of sentence.
  l. Line 253- Please re-write this sentence to make it clearer.
  li. Line 255- Use “significantly” instead of “statistically”
  lii. Line 255-260- This sentence does not give a clear sense. Please re-write.

# COMMENTS ON THE MAIN PAPER

## Abstract

- Line 29 – The author has used both bivariable and multivariable logistic regression to determine the independent predictors. Please use “Binary and multivariable logistic regression analyses.”
- Line 32 ( Result Section)- This is not a causal model of analysis. So, it would be better to say “Adjusting for other variables” instead of using the word confounders.
- Line 37 ( Conclusion)- Please re-write the sentence “it needs to deal with awareness”. Here “it” is a vague word. Please clarify what the author wants to mention.

## Introduction

- Line 57- Please re-write the sentence *“Breast cancer is not only a disease of the developed countries although low and middle-income countries are also affected.”*

## Materials and Methods

- Line 85- Please justify why the author used the 1.5 design effect.
- Line 141- Please use the word “ bivariable” instead of bivariate. Also it would be better to use words uniformly; either “binary logistic regression”, or “bivariable logistic regression” in all the sections of the paper.
- Line 200- Please use the word “multivariable analysis/regression” throughout the manuscript instead of the word “multivariate analysis/regression”. Please see the references for your reading . From one of the papers below-“*Statistically speaking, multivariate analysis refers to statistical models that have 2 or more dependent or outcome variables, and multivariable analysis refers to statistical models in which there are multiple independent or response variables.”*

<https://www.karger.com/article/FullText/345491#:~:text=The%20terms%20'multivariate%20analysis'%20and,outcome%20each%20time%20%5B1%5D>.

<https://www.ncbi.nlm.nih.gov/pmc/articles/PMC3518362/>

- Line 142- Please clarify why the author used the p-value of 0.25 instead of 0.05 in the bivariable model.
- Also, please be consistent with the use of the p-value of 0.25 or 0.05, as the p-value of 0.05 has been indicated in line 366 of Table 3 ( Bivariable Analysis).
- Line 142-144- Please elaborate on why the author used the method “Back ward likelihood ratio of logistic regression”.
- Line 145- “AOR” stands for Adjusted Odds Ratio, not just Odds Ratio.
- Line 147-148- Please clearly indicate if this statement *“P-value < 0.05 was considered as statistically significant in the study”* is for **multivariable model.**
- According the to the STROBE checklist, all the VARIABLES should be clearly defined in the Method Section.
  - The outcome variable is the division of respondent’s score by the mean score. This sounds a bit arbitrary – what would happen if the mean is skewed because of outlier values? The usual method is to use the median – using quantile regression as the multivariable method. Please clarify on this.
  - The knowledge score is not well defined – what were the questions that went into the score? How much was the scale and what were the minimum and maximum? All these need to be part of the methods description of the variables.
  - Please Insert a VARIABLE Section and define all the variables clearly.

## Result

- Please check the tense in this section as indicated in General Comments section.
- Line 196- Please be consistent with the use of the p-value of 0.25 or 0.05, as it is indicated p-value of 0.05 in line 366 of Table 3 ( Bivariable Analysis).
- Line 201-203: It would be better to include these sentences in the Data Analysis Section.
- In the Result Section, as well as in the Tables, two significant digits after the decimal point are enough.

## Discussion

- Line 222-224- *“This difference might be due to sample size, respondent’s inclusion criteria, and data collection methods, total number of items they were used to ask the respondents, construction and compute of the awareness item”*. The author could specify one or two instances of how the different methodological aspects like sample size or total number of items used to ask respondents could have led to lower prevalence of breast cancer awareness in Eastern China (18.6%) and Saudi Arabia (39.7%) , as compared to this study (64.3%).
- According to Strobe Checklist, there needs to be a section with LIMITATIONS and INTERPRETATION in the discussion. Please add that.

## Conclusion

- This section requires re-writing as indicated in the General Comments Section.

# COMMENTS ON THE TABLE

## Table 1

- Check the case ( higher/lower) of the letters of the variables

## Table 2

- The author put asterisk sign in the footnote as *“*more than one option is reported by a participant”*, but the asterisk sign is not indicated anywhere in the table.

## Table 3 and Table 4

- The author could show the information in Table 3 and Table 4 ( Crude Odds Ratio and Adjusted Odds Ratio) in a single table for better presentation and clarity.
- Line 366- *“ Note: * p value of less than 0.05 in bivariate analysis”.* The author had described in the Method Section (Line 142) that in the bivariable analysis, they used the p-value of 0.25. In this Line 366, they indicate p value of 0.05. Please apply consistent methods, and clarify.
- Line 366- Also check the appropriate use and label of asterisk sign “ * ” defined as “*p value of* ***less than 0.05*** *in bivariate analysis”* , since this asterisk sign has also been used in “<0.001*”.
