# Supplementary material for: Assessing breast cancer awareness on reproductive age women in West Badewacho Woreda, Hadiyya Zone, South Ethiopia; Community based cross- sectional study
Source: PLoS One. 2022 Jul 27;17(7):e0270248. doi: 10.1371/journal.pone.0270248 (PMC9328547; doi:10.1371/journal.pone.0270248)
Supplement: Attachment — Submitted filename: response to editor and reviewer.docx [file pone.0270248.s004.docx]

Response to editor and reviewers

Dear Editor and revewers

Thank you very much for your consideration of our manuscript and constructive, valuable and educational comments. We have considered each of them, and provide the changes affected below in turn. Where changes have not been made, we provide reasons for this to substantiate our view.

Thanks, we were rewriting our manuscript as you told.

Thanks, we also elaborated study design and design effect on the manuscript.

We used design effect for we have woreda, then kebeles, lastly gotti(mahandari). So, we have at least two stages. So, we used design effect.

1. Please ensure that your manuscript meets PLOS ONE's style requirements, including those for file naming. The PLOS ONE style templates can be found at
   [https://journals.plos.org/plosone/s/file?id=wjVg/PLOSOne_formatting_sample_main_body.pdf](https://www.google.com/url?q=https%3A%2F%2Fjournals.plos.org%2Fplosone%2Fs%2Ffile%3Fid%3DwjVg%2FPLOSOne_formatting_sample_main_body.pdf&sa=D&sntz=1&usg=AFQjCNGe6oxB056ucvyxvHe8qob9S8_XkA) and 
   [https://journals.plos.org/plosone/s/file?id=ba62/PLOSOne_formatting_sample_title_authors_affiliations.pdf](https://www.google.com/url?q=https%3A%2F%2Fjournals.plos.org%2Fplosone%2Fs%2Ffile%3Fid%3Dba62%2FPLOSOne_formatting_sample_title_authors_affiliations.pdf&sa=D&sntz=1&usg=AFQjCNHjeuwjv87FpHKY5ilTaf_n-Rp8PQ).

Thank you

- We have updated the style, layout and naming of manuscript in line with the requirements of PLOS ONE.

2. In your Methods section, please provide additional information about the participant recruitment method and the demographic details of your participants. Please ensure you have provided sufficient details to replicate the analyses such as: a) the recruitment date range (month and year), b) a description of any inclusion/exclusion criteria that were applied to participant recruitment.

Thank you

- All your requirements were incorporated in the manuscript like participants recruitments methods, recruitment date & range, inclusion and exclusion criteria.

3. Please ensure you have discussed any potential limitations of your study in the Discussion, including study design, sample size and/or potential confounders.

Limitation parts was added and considered.

4. Thanks a lot.

In the Methods, please state:
- Why written consent could not be obtained
- Whether the Institutional Review Board (IRB) approved use of oral consent
- How oral consent was documented

- This entire question was addressed in manuscript part in detail. Our subjects were human. So, ethical part was applied as research ethics guideline

5. a) Please clarify the sources of funding (financial or material support) for your study. List the grants or organizations that supported your study, including funding received from your institution.
   b) State what role the funders took in the study. If the funders had no role in your study, please state: “The funders had no role in study design, data collection and analysis, decision to publish, or preparation of the manuscript.”
   c) If any authors received a salary from any of your funders, please state which authors and which funders.
   d) If you did not receive any funding for this study, please state: “The authors received no specific funding for this work.”

- Thanks, we have considered these parts. Our funders were the institution but have no role in study design, data collection and analysis, decision to publish, or preparation of the manuscript

6. Please complete your Competing Interests on the online submission form to state any Competing Interests. If you have no competing interests, please state "The authors have declared that no competing interests exist.", as detailed online in our guide for authors at [http://journals.plos.org/plosone/s/submit-now](http://www.google.com/url?q=http%3A%2F%2Fjournals.plos.org%2Fplosone%2Fs%2Fsubmit-now&sa=D&sntz=1&usg=AFQjCNFeMGrMjJ9uhUwWGbg8DgPACiSGSQ)
    This information should be included in your cover letter; we will change the online submission form on your behalf.

- Thank u, we also consider as your recommendation. So, the authors have declared that no competing interests exist.

7. We note that you have stated that you will provide repository information for your data at acceptance. Should your manuscript be accepted for publication, we will hold it until you provide the relevant accession numbers or DOIs necessary to access your data. If you wish to make changes to your Data Availability statement, please describe these changes in your cover letter and we will update your Data Availability statement to reflect the information you provide.

- Thank u. we will provide full data information for you, if you want any time.

8. Please upload a copy of Figure 1, to which you refer in your text on page 18

- Thank u. this was corrected in manuscript. It was figure 2.

9. Please ensure that you refer to Figure 3 in your text as, if accepted, production will need this reference to link the reader to the figure.

- Also this part was corrected as figure 2. It was corrected in manuscript part

10. Please include captions for your Supporting Information files at the end of your manuscript, and update any in-text citations to match accordingly. Please see our Supporting Information guidelines for more information: [http://journals.plos.org/plosone/s/supporting-information](http://www.google.com/url?q=http%3A%2F%2Fjournals.plos.org%2Fplosone%2Fs%2Fsupporting-information&sa=D&sntz=1&usg=AFQjCNGy726HD6dB6aDPQ8yeHTY7vFV8lA).

Thanks a lot. We were considering it.

11. We noticed you have some minor occurrence of overlapping text with the following previous publication(s), which needs to be addressed:

- All this part was considered in manuscript. A manuscript was rewrite and cites all references.

Reviewer 1

Really we have great appreciation to your constructive and valuable comments and suggestion. In general, we have modified our manuscript completely as your comments and suggestion. For seek response we have some reaction on your comments

1. The author states that since breast cancer awareness is not well-documented among Ethiopian women, this study assesses awareness of the breast cancer and associated factors among reproductive-aged women in Hadiya Zone, West Badewacho Woreda of Ethiopia.

**e.g. According to WHO report 2020**

Cancer is a leading cause of death worldwide, accounting for nearly 10 million deaths in 2020 (1). The most common in 2020 (in terms of new cases of cancer) were:

- **breast (2.26 million cases);**
- lung (2.21 million cases);
- colon and rectum (1.93 million cases);
- prostate (1.41 million cases);
- skin (non-melanoma) (1.20 million cases); and
- stomach (1.09 million cases).

Between 30 and 50% of cancers can currently be prevented by avoiding risk factors and implementing existing evidence-based prevention strategies. The cancer burden can also be reduced through early detection of cancer and appropriate treatment and care of patients who develop cancer. Many cancers have a high chance of cure if diagnosed early and treated appropriately.

- *This means that a little known about breast cancer especially in rural women. Because of this they seek care in late stage of cancer and even some of them didn’t understand it was disease or not. Also, in our study area there was no study conducted. So, developing mother’s awareness to this disease may develop women to treat early and manage timely.*

2. Some of the methodological parts have not been clearly described, and there are some inconsistencies, as suggested in the “Materials and Methods” section below.

- *Thank u. this part was considered. We made it clear and consistent in manuscript as your comments.*

3. Some elaborations and clarifications can be made in the Discussion section, as suggested in the “Discussion” section below.

- *This has been revised according to your suggestion.*

Abstract part

- Line 29 – The author has used both bivariable and multivariable logistic regression to determine the independent predictors. Please use “Binary and multivariable logistic regression analyses.”

Thank u. It is considered in manuscript part as bivariable and multivariable analysis. For general knowledge binary logistic regression is used for categorical varibles especially dependent variable is dichotomies.

- Line 32 ( Result Section)- This is not a causal model of analysis. So, it would be better to say “Adjusting for other variables” instead of using the word confounders. This part is also considered in manuscript as you suggested.
- Line 37 ( Conclusion)- Please re-write the sentence “it needs to deal with awareness”. Here “it” is a vague word. Please clarify what the author wants to mention.

This was also considered in manuscript and re-written.

## Introduction

- Line 57- Please re-write the sentence *“Breast cancer is not only a disease of the developed countries although low and middle-income countries are also affected.”*

*This part is considered in manuscript as you suggest.*

## Materials and Methods

- Line 85- Please justify why the author used the 1.5 design effect.

*Thank u my reviewer. we used at least two stages lower in the sampling process to arrive at the final sampling unit. As a result, we used a design effect of 1.5 to multiply our sample size in order to minimize variability and detect the effect observed regarding breast cancer awareness.*

- Line 141- Please use the word “ bivariable” instead of bivariate. Also it would be better to use words uniformly; either “binary logistic regression”, or “bivariable logistic regression” in all the sections of the paper.

*It was considered as you suggested. Generally, bivariable for one dependent variable with one independent variable, for multivariable dependent variable with two or more independent variables.*

- Line 200- Please use the word “multivariable analysis/regression” throughout the manuscript instead of the word “multivariate analysis/regression”. Please see the references for your reading . From one of the papers below-“*Statistically speaking, multivariate analysis refers to statistical models that have 2 or more dependent or outcome variables, and multivariable analysis refers to statistical models in which there are multiple independent or response variables.”*

<https://www.karger.com/article/FullText/345491#:~:text=The%20terms%20'multivariate%20analysis'%20and,outcome%20each%20time%20%5B1%5D>.

<https://www.ncbi.nlm.nih.gov/pmc/articles/PMC3518362/>

*-This part is also corrected as you suggest*

- Line 142- Please clarify why the author used the p-value of 0.25 instead of 0.05 in the bivariable model.

*As you know that bivariable analysis is one outcome variable with one explanatory variable analysis. So, we used p-value <0.25 to get important variables that predict outcome variable due to bivariate analysis only compute with one variable. So, increase candidate variables for multivariable analysis. This mostly determine true predictor for outcome variable. As convection use criteria to select variables in bivariable analysis.*

- Also, please be consistent with the use of the p-value of 0.25 or 0.05, as the p-value of 0.05 has been indicated in line 366 of Table 3 ( Bivariable Analysis).

*Thank u. we used consistent throughout manuscript as your suggestion. But we used p-value <0.25 for bivariable analysis and p-value <0.05 for multivariable analysis.*

- Line 142-144- Please elaborate on why the author used the method “Back ward likelihood ratio of logistic regression”.

*Thank u. why we used this backward likelihood ratio of logistic regression from others types of stepwise due it has better than other like Starting with the full model has the advantage of considering the effects of all variables simultaneously. Also, important in case of collinearity. Due to force to keep them all in the model.*

- Line 145- “AOR” stands for Adjusted Odds Ratio, not just Odds Ratio.

*It was considered in manuscript part*

- Line 147-148- Please clearly indicate if this statement *“P-value < 0.05 was considered as statistically significant in the study”* is for **multivariable model.**

*Yes. It was considered in manuscript as you suggest*

- According the to the STROBE checklist, all the VARIABLES should be clearly defined in the Method Section.
  - The outcome variable is the division of respondent’s score by the mean score. This sounds a bit arbitrary – what would happen if the mean is skewed because of outlier values? The usual method is to use the median – using quantile regression as the multivariable method. Please clarify on this.

*Thank u. we used distribution of the outcome variable before mean score calculation. So, our data was normally distributed.*

- The knowledge score is not well defined – what were the questions that went into the score? How much was the scale and what were the minimum and maximum? All these need to be part of the methods description of the variables.

*It was considered in manuscript part specifically in operational definition part*

- Please Insert a VARIABLE Section and define all the variables clearly.

*It was inserted in manuscript part as you suggested.*

## Result

- Please check the tense in this section as indicated in General Comments section.

*It was checked and re arranged in document.*

- Line 196- Please be consistent with the use of the p-value of 0.25 or 0.05, as it is indicated p-value of 0.05 in line 366 of Table 3 ( Bivariable Analysis).

*Thanks a lot. We used and try to clarify in above section we used p-value <0.25 in bivariate analysis to get candidate variables and p-value <0.05 for multivariable analysis. But, we use convection and rule of tump specially in bivariable analysis.*

- Line 201-203: It would be better to include these sentences in the Data Analysis Section.

*It was considered in document*

- In the Result Section, as well as in the Tables, two significant digits after the decimal point are enough.

*This part was also considered in document as you recommended*

## Discussion

- Line 222-224- *“This difference might be due to sample size, respondent’s inclusion criteria, and data collection methods, total number of items they were used to ask the respondents, construction and compute of the awareness item”*. The author could specify one or two instances of how the different methodological aspects like sample size or total number of items used to ask respondents could have led to lower prevalence of breast cancer awareness in Eastern China (18.6%) and Saudi Arabia (39.7%) , as compared to this study (64.3%).

*Prevalence -The number of cases of a disease (or people with a particular characteristic) existing in a specified population at a given point in time. So, if sample size decrease cases probability to get cases might be decrease. Also, item they used to ask respondents different from us or categorization way may influence prevalence due to understandability of item to respondents affect responses to item. This mean that item number increase the clarity to answer increase.*

- According to Strobe Checklist, there needs to be a section with LIMITATIONS and INTERPRETATION in the discussion. Please add that.

*Considered and incorporated in manuscript part especially limitation. But interpretation was incorporated in conclusion part.*

## Conclusion

- This section requires re-writing as indicated in the General Comments Section.

This section was re-written in manuscript as you suggested.

# COMMENTS ON THE TABLE

## Table 1

- Check the case ( higher/lower) of the letters of the variables

*It was checked and corrected in manuscript.*

## Table 2

- The author put asterisk sign in the footnote as *“*more than one option is reported by a participant”*, but the asterisk sign is not indicated anywhere in the table.

*This part was corrected and asterisk was putted in correct place on table as you mentioned.*

## Table 3 and Table 4

- The author could show the information in Table 3 and Table 4 ( Crude Odds Ratio and Adjusted Odds Ratio) in a single table for better presentation and clarity.

*Considered and merged in one table as your suggestion*

- Line 366- *“ Note: * p value of less than 0.05 in bivariate analysis”.* The author had described in the Method Section (Line 142) that in the bivariable analysis, they used the p-value of 0.25. In this Line 366, they indicate p value of 0.05. Please apply consistent methods, and clarify.

*It was mentioned above why we used p-value less than 0.25 and p- value less than 0.05.*

- Line 366- Also check the appropriate use and label of asterisk sign “ * ” defined as “*p value of* ***less than 0.05*** *in bivariate analysis”* , since this asterisk sign has also been used in “<0.001*”.

*Why we used <0.001*, for those p-value was 0.000 and to show strong relationship between dependent and independent variables.*

**Reviewer 2**

Really we have great appreciation to your constructive and valuable comments and suggestion. In general, we have modified our manuscript completely as your comments and suggestion
