# Supplementary material for: Assessing breast cancer awareness on reproductive age women in West Badewacho Woreda, Hadiyya Zone, South Ethiopia; Community based cross- sectional study
Source: PLoS One. 2022 Jul 27;17(7):e0270248. doi: 10.1371/journal.pone.0270248 (PMC9328547; doi:10.1371/journal.pone.0270248)
Supplement: Attachment — Submitted filename: Response to reviewer 3.docx [file pone.0270248.s005.docx]

Response to reviewer

Dear Peer Reviewer,

Thank you very much still for your consideration of our manuscript and constructive, valuable and educational comments. We have considered each of them, and provide the changes affected below in turn. Where changes have not been made, we provide reasons for this to substantiate our view.

Really, we have great appreciation to your constructive and valuable comments and suggestion. In general, we have modified our manuscript completely as your comments and suggestion. For seek response we have some reaction on your comments

Result part

1. Remove FIGURE 2 AWARENESS OF BREAST CANCER AMONG REPRODUCTIVE-AGE WOMEN IN WEST BADEWACHO WOREDA, HADIYA ZONE, SOUTH ETHIOPIA,2020 (N=364). Describe it in text.

- *Thank u. this part was considered as you commented.*

2. FIGURE 3 SOURCE OF INFORMATION FOR THE AWARENESS, present a simple bar diagram.

- *This has been revised according to your suggestion.*

**Discussion**
Revise the discussion section, focused more on what is the existing health promotion program in the country. How this study findings will be useful to promote breast cancer screening in the country.

*Thank u. we were discussed most of our findings with other findings conducted in other part of countries. As result we focused on contemporary issues. As you know that if women aware for breast cancer, they will be screened for breast cancer regularly. if not, the chance of screening is low. our aim was to identify level of awareness of reproductive aged women rather than screening awareness. Some howe it was raised on significant of study part and conclusion.*
